# The Impact of Hempseed Consumption on Bone Parameters and Body Composition in Growing Female C57BL/6 Mice

**DOI:** 10.3390/ijerph19105839

**Published:** 2022-05-11

**Authors:** Cynthia A. Blanton, Jared J. Barrott, Kaden Kunz, Ella Bunde, Hailey M. Streff, Chandler A. Sparks, Derrick W. Williams, Annette M. Gabaldόn

**Affiliations:** 1 Department of Nutrition and Dietetics, Idaho State University, Pocatello, ID 83209, USA; 2 Department of Biomedical and Pharmaceutical Sciences, Idaho State University, Pocatello, ID 83209, USA; jaredbarrott@isu.edu (J.J.B.); kadenkunz@isu.edu (K.K.); ellabunde@isu.edu (E.B.); 3 Department of Biology, Colorado State University-Pueblo, Pueblo, CO 81001, USA; hm.streff@pack.csupueblo.edu (H.M.S.); ca.sparks@pack.csupueblo.edu (C.A.S.); annette.gabaldon@csupueblo.edu (A.M.G.); 4 Harvard Medical School, Boston, MA 02115, USA; dwilliams@hms.harvard.edu

**Keywords:** hempseed, bone, osteoclasts, osteoblasts, C57BL/6 mice, polyphenols, polyunsaturated fatty acids, DEXA

## Abstract

Optimizing peak bone mass is critical to healthy aging. Beyond the established roles of dietary minerals and protein on bone integrity, fatty acids and polyphenols modify bone structure. This study investigated the effect of a diet containing hempseeds (HS), which are rich in polyunsaturated fatty acids and polyphenols, on bone mineral density, bone cell populations and body composition. Groups (*n* = 8 each) of female C57BL/6 mice were fed one of three diets (15% HS by weight; 5% HS; 0% HS (control)) from age 5 to 30 weeks. In vivo whole-body composition and bone mineral density and content were measured every 4 weeks using dual-energy X-ray absorptiometry. Ex vivo humeri cell populations in the epiphyseal plate region were determined by sectioning the bone longitudinally, mounting the sections on slides and staining with tartrate-resistant acid phosphatase and alkaline phosphatase stain to identify osteoclasts and osteoblasts, respectively. Mixed models with repeated measures across experimental weeks showed that neither body weight nor body weight gain across weeks differed among groups yet mice fed the 15% HS diet consumed significantly more food and more kilocalories per g body weight gained than those fed the 5% HS and control diets (*p* < 0.0001). Across weeks, fat mass was significantly higher in the 5% HS versus the control group (*p* = 0.02). At the end point, whole-body bone mineral content was significantly higher in the control compared to the 5% HS group (*p* = 0.02). Humeri from both HS groups displayed significantly lower osteoblast densities compared to the control group (*p* < 0.0001). No relationship was seen between osteoblast density and body composition measurements. These data invite closer examination of bone cell activity and microarchitecture to determine the effect of habitual HS consumption on bone integrity.

## 1. Introduction

Osteoporosis is among the most costly diseases of aging, imposing severe consequences on quality of life and health care expenditures [1]. Prevention of osteoporosis is key considering the modest effect of current treatments, and a primary means of prevention is to maximize bone accrual during development [2,3]. Dietary recommendations for achieving and maintaining bone mass include adequate intakes of calcium and vitamin D [4], yet ~50% and 95%, respectively, of Americans fail to meet the requirements [5]. Other dietary constituents, such as fatty acids and polyphenols, impact bone integrity, as indicated by epidemiological, animal and in vitro studies [6,7,8,9,10]. However, this function of fatty acids and polyphenols is understudied and incompletely understood in the context of bone health, likely due to research emphasis on the association of dietary fat and oxidative stress to cardiovascular disease [11,12]. The lack of recognition of these nutrients as bone-building compounds represents a missed opportunity for public health.

Characterizing the impact and mechanisms of fatty acids and polyphenols in bone development is important to expanding the range of bone-protective interventions. Polyunsaturated fatty acids (PUFAs), including the essential omega-3 fatty acid alpha-linolenic acid (ALA), and polyphenols are concentrated in plant foods such as seeds, and consumption of these dietary components is positively associated with bone mass in adults [13,14,15,16,17,18,19,20]. The growing public interest in plant-based diets and healthful fats positions consumption of seed products as an attractive food choice for consumers. Further, plant sources of ALA and other PUFAs are more likely than fish to be included in the diets of many people and, thus, this area of research could translate into effective, attainable dietary guidelines for bone health.

The mechanisms by which fatty acids and polyphenols modulate bone structure are not fully understood and existing data have been produced primarily from animal and cell culture experiments [7,9,10,21,22,23,24,25]. The mechanisms identified include: (1) modulation of prostaglandin synthesis with subsequent inhibition of osteoclast formation and differentiation [10,21], (2) regulation of bone morphogenic protein release and osteoblast activity [26,27], (3) modulation of inflammatory signaling pathways [23,28] and (4) intestinal microbiota fermentation of hempseed fiber to short-chain fatty acids [22,29]. Lacking are data pertaining to the effect of long-term consumption of omega-3 fatty acid + polyphenol-rich food on bone cell types and bone structure during skeletal development. Given the evidence supporting an effect of fatty acids and polyphenols on bone integrity and immune system function [24,30,31], an important next step is to determine whether long-term consumption of a seed-enriched diet (high in omega-3 fatty acids and polyphenols) versus a control diet during development improves bone structure in association with bone cell populations that are consistent with bone accretion (higher numbers of bone-building osteoblasts and lower numbers of bone-destroying osteoclasts).

Characterizing bone cells may uncover mechanisms by which dietary seed impacts bone. Bone is a dynamic tissue marked by continuous cell turnover [32]. Stem cells originate in bone marrow and proceed to follow distinct differentiation pathways. Two cell types that develop from marrow precursor cells are osteoclasts, which degrade bone, and osteoblasts, which rebuild new bone in the cavities created by osteoclasts. Early life is a period when bone accretion occurs due to greater osteoblast versus osteoclast activity. Immune cells, which also originate in marrow, influence osteoblast/clast activity. Specifically, immune cell proliferation increases the production of reactive oxygen species that promote osteoclastic degradation of bone.

Another factor influencing bone integrity is marrow adipose content. Evidence supports that diets rich in omega-3 fatty acids versus omega-6 fatty acids interfere with adipose accumulation in bone marrow with age [23,25]. This is an important aspect of bone composition to investigate in growing mice since excess adipose tissue in bone marrow reduces bone strength and is associated with the development of osteoporosis [23,33,34].

No known studies have investigated the effect of long-term feeding of a seed-enriched diet on bone health in a mammalian species. A short-term (35 days) feeding trial in cockerels showed increased tibia strength after dietary enrichment with hempseed + rapeseed compared to a control diet [35]. A longer-term (12 weeks) experiment in hens also demonstrated increased tibia strength with diets containing a range of hempseed concentrations [36]. Hempseed (*Cannabis sativa* L.) production has recently been expanded in the United States [37], generating increased interest in hemp research and marketing [38,39]. Hempseed contains a high omega-3 to omega-6 fatty acid ratio and a high concentration of antioxidants and polyphenols that support the potential of hempseed as a dietary intervention for optimizing bone health [19,40]. The objectives of this study were to examine the effect of hempseed-rich diets on whole-body composition and skeletal bone parameters in C57BL/6 female mice. In vivo studies included dual-energy X-ray absorptiometry scanning at monthly intervals over 25 weeks while in vitro studies included immunohistochemistry for detection of bone cell densities in the humerus at endpoint.

## 2. Materials and Methods

### 2.1. Experimental Design

The experiment was performed in accordance with the Guide for the Care and Use of Laboratory Animals composed by the National Institutes of Health. The use of animals and the study protocol were approved by the Colorado State University-Pueblo, USA Institutional Animal Care and Use Committee under protocol number 000-000A-021. Female C57BL/6 mice of age 3 weeks were purchased from Charles River Laboratories (Wilmington, MA, USA) and acclimated to the facility for 2 weeks, during which time they were fed a pellet control diet (AIN-93G). Mice were housed in pairs in one environmentally controlled room with lights on at 0600 and off at 1800 h. Each mouse was implanted subcutaneously in the subscapular region with a programmable microchip (UID Identification Solutions, Lake Villa, IL 60046, USA; www.uidevices.com (accessed on 7 May 2022)) at age 4 weeks. At age 5 weeks, mice were separated into groups (*n* = 8 each) and fed one of three diets (15% hempseed (HS) by weight; 5% HS; 0% HS (control)) until age 30 weeks. The age range for the mice was chosen as it correlates to childhood through young adulthood in humans and encompasses the period when peak bone mass is achieved [41]. Water and food were provided ad libitum. From weeks 5–30, body weight and food intake were measured weekly and body composition and bone mineral content and density were measured monthly.

### 2.2. Diets

Diets were based on the AIN-93G diet [42] and purchased from Dyets, Inc. (Bethlehem, PA, USA) in pellet form. The control diet was unmodified AIN-93G. Two hempseed diets were created by including 5% or 15% by weight whole ground, organic, toasted hempseed (CHII Naturally Pure Hemp—Naturally Splendid Enterprises, Ltd., Pitt Meadows, BC, Canada). The composition of the diets is shown in Table 1. Diets contained ~16% of total kcals from fat. Where there are adjustments to the base diet, it is to account for nutrients present naturally in whole hempseed. For example, cellulose is reduced from 50 g/kg diet (control) to 34.5 g/kg diet (5% HS) and 3.5 g/kg diet (15% HS) to account for the fiber contributed by the hempseed. Nutrition information for the hempseed is shown in Supplementary Table S1.

### 2.3. Body Weight and Food Intake Measurement

Body weight was measured for individual mice at the same time of day once a week using a digital scale. Food intake was determined for each mouse of a co-housed pair by measuring the food cup in and out weight once a week and dividing by 2.

### 2.4. Body Composition and Bone Mineral Measurement

Body composition and bone mineral content and density were measured monthly by dual-energy X-ray absorptiometry using a Lunar PIXImus scanner (GE Lunar, Madison, WI, USA). Mice were anesthetized with 2.5% isoflurane and placed in the prone position on the specimen tray. The skull was excluded from analysis. Measurements collected included fat mass (g), lean mass (g), bone mineral content (g), and bone mineral density (g/cm^2^).

### 2.5. Determination of Bone Cell Density and Epiphyseal Plate Thickness

At the conclusion of the feeding experiment, mice were euthanized using 4% isoflurane followed by cervical dislocation and right humeri were dissected. Dissected humeri were immediately fixed with a cross linker (4% paraformaldehyde in phosphate-buffered saline) for 24 h. Bones were then transferred to 14% EDTA and stored for 3 weeks for decalcification.

After decalcification, the humeri were dehydrated with an ethanol series. Bones were embedded in paraffin and sectioned longitudinally on a microtome. The bones were sectioned until the marrow was exposed and sections of 5 µm thickness were cut and mounted on slides. Slides were dewaxed using SafeClear (Fisher Scientific, Hampton, NH, USA) and rehydrated in an ethanol series of 100% to water. To evaluate the difference in densities between osteoblasts and osteoclasts in the distal epiphyseal region, the paraffin-embedded tissues were stained on the slides using tartrate-resistant acid phosphatase stain (TRAP) and the alkaline phosphatase stain (ALP). ALP stains for osteoblasts and TRAP stains for osteoclasts [43,44]. Slides were analyzed by 5 different evaluators; 3 were blind to the treatment conditions. Sixteen slides total (one per mouse) were ranked for the amount of stain activity by determining the intensity of purple stain at the distal epiphyseal plate. Two samples were not included due to a lack of an epiphyseal plate in the hematoxylin and eosin (H&E) stain. The remaining 14 slides (*n* = 4, control; *n* = 6, 5% HS; *n* = 4, 15% HS) were ranked from high to low (14, high and 1, low) and were used in the analysis.

**Figure 1:**
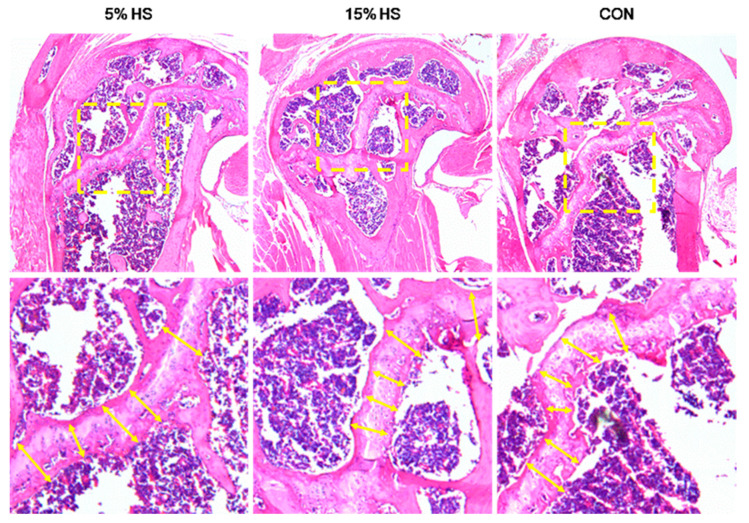
H&E-stained sections of the humerus distal epiphyseal plate from mice representing the three treatment groups (5% HS, 15% HS and control). Top row: Yellow dashed line boxes show the region of interest. Bottom row: Yellow arrows indicate the positioning of markers of measurements of growth plate thickness. For each image, only 5–6 markers (of 20 total per sample) are shown to better visualize the growth plate.

Using the same H&E slides, images were captured with the 5X objective on a Leica DM6B widefield microscope and imaged with the attached Leica DFC450-C digital camera. Digital images were analyzed for thickness (μm) of the distal epiphyseal plate using FIJI software by taking 20 measurements along the epiphyseal plate for each sample using a perpendicular angle between the edges of the growth plate as indicated by the yellow arrows in Figure 1. Technical replicates were averaged together to provide the number for each biological sample. Statistical analysis was performed using the biological replicates.

### 2.6. Immunohistochemistry Determination of Bone Marrow Adipocyte Density

To examine the effect of dietary hempseed on the density of bone marrow adipocytes, immunohistochemistry staining for the marker PRDM16 protein was employed. This zinc finger transcription factor plays an important role in the differentiation of adipocytes within the bone marrow [45,46,47]. Tissue was stained using a rabbit anti-human polyclonal antibody targeting PRDM16 (ThermoFisher Scientific, Waltham, MA, USA; catalog #PA5-20872). All other reagents were obtained from the Pierce Peroxidase Detection Kit (ThermoFisher Scientific; catalog #36000) and the protocol was followed according to the manufacturer’s instructions. Briefly, a goat anti-rabbit secondary antibody was applied to the tissues, followed by an anti-goat strep-HRP tertiary antibody. Samples were counterstained with hematoxylin, dehydrated and mounted. Slides were analyzed for PRDM16-positive cells (staining intensity) using a Leica DM6B widefield microscope and imaged with the attached Leica DFC450-C digital camera. Quantitative values were obtained by counting PRDM16-positive cells (brown stain) and total cells (blue stain) in the bone marrow and calculating a percentage.

### 2.7. Dual-Energy X-ray Absorptiometry Analysis of Humerus Bone BMC and BMD at Distal Region

**Figure 2:**
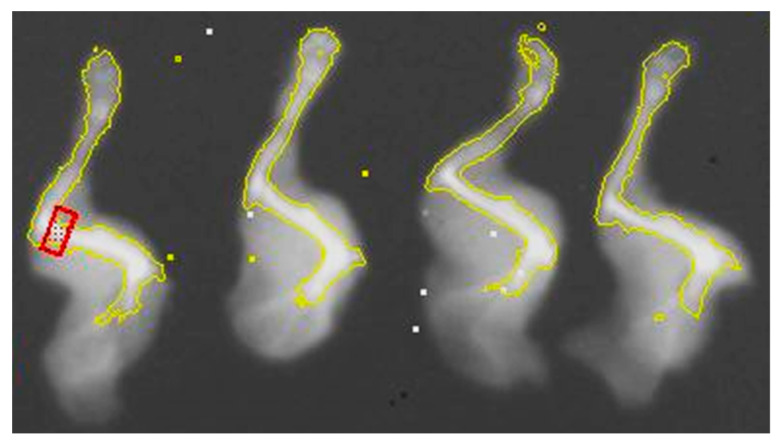
Example of humerus bone distal region dual-energy X-ray absorptiometry analysis. Mouse dissected left forelimbs were scanned in groups of *n* = 4. Images were adjusted for high contrast. A small ROI (red box) was placed at the distal end of the humerus bone in the approximate region where bone densities and epiphyseal plate thickness were measured. Site-specific BMC and BMD were then analyzed.

Site-specific measurements of humerus bone BMC and BMD at endpoint (age 30 weeks) were also made to correlate with bone cell density data. The contralateral (left) forelimb was dissected and dual-energy X-ray absorptiometry scanned (Figure 2). For each scan, a set of four limbs was placed on an “artificial tissue block” and scanned, then images were adjusted for high contrast. A small ROI (red box) was placed at the distal end of the humerus bone in the approximate region where bone densities and epiphyseal plate thickness were measured.

### 2.8. Statistical Analysis

Data were analyzed using JMP Pro 16 statistical software (Cary, NC, USA). Data were assessed for normal distribution and equality of variances using the Shapiro–Wilk test and analysis of means for variances with Levene test, respectively. Data that were not normally distributed were transformed to log normal prior to analyses. One-way analysis of variance (ANOVA) followed by Tukey–Kramer HSD post hoc multiple comparisons were used to compare group means when variances were equal. Nonparametric one-way tests followed by Wilcoxon nonparametric comparisons were used when variances were not equal. ANOVA with repeated measures was used to compare the effect of diet group on body weight, body composition and food intake over the weeks of the experiment. Linear regression analysis and one-way ANOVA were used to determine the relationship between endpoint in vivo bone mineral measurements and ex vivo humeri osteoblast density. A Chi-square test with a Yates correction was used to compare the observed and expected frequencies of PRDM16-positive cells in the bone marrow of mice from the various treatment groups. Descriptive data are reported as means with standard deviations. Figures with trendlines were created using Graph Builder in JMP Pro.

## 3. Results

### 3.1. Body Weight and Food Intake

**Figure 3:**
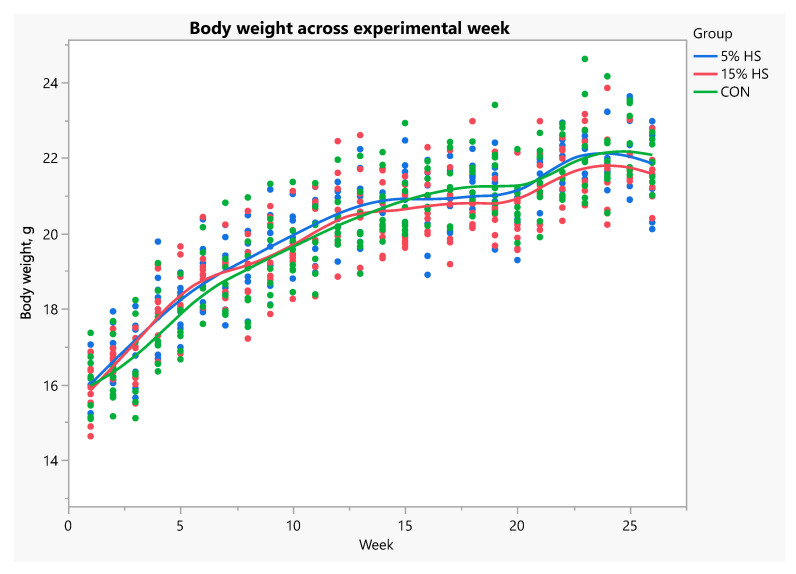
Body weight measured at week’s end across experimental weeks. N = 8 per group. Each dot represents an individual mouse; colors represent the experimental diet group. Mixed models with repeated measures showed no significant differences across groups (*p* = 0.19). Effect of week, *p* < 0.0001.

**Figure 4:**
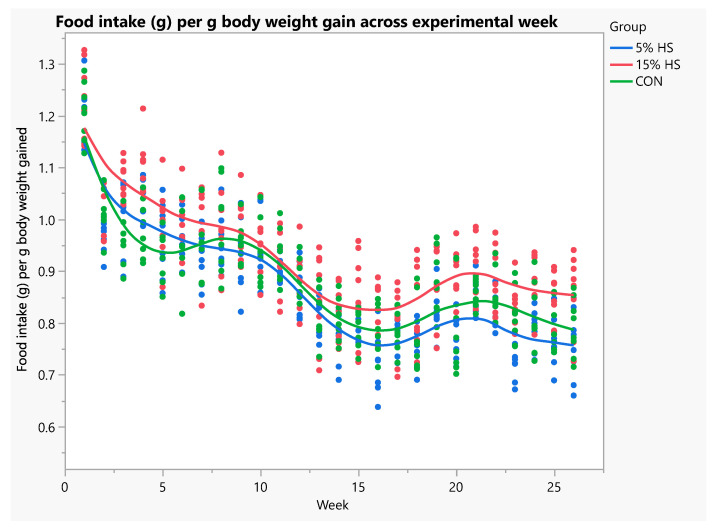
Food intake (g) consumed per g body weight gained across experimental week. N = 8 per group. Each dot represents an individual mouse; colors represent the experimental diet group. Mixed models with repeated measures showed a significant effect of group, with 15% HS consuming significantly more food than the 5% HS and control groups (*p* < 0.0001). Effect of week, *p* < 0.0001.

**Figure 5:**
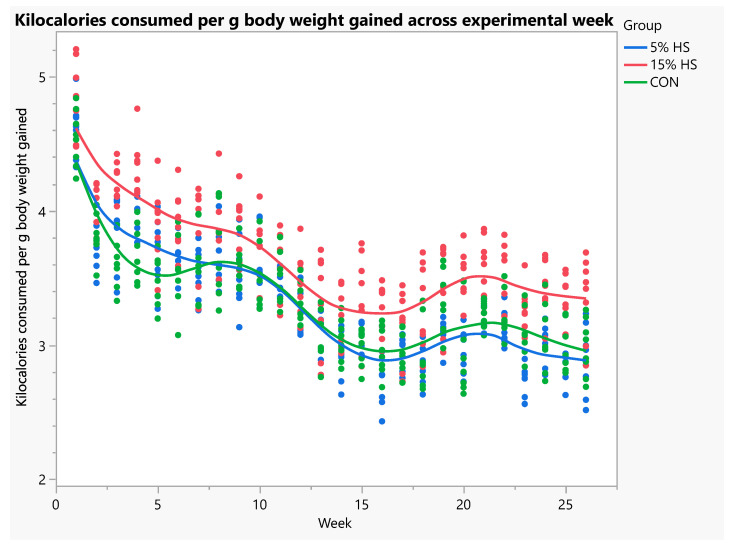
Kilocalories consumed per g body weight gained across experimental weeks. N = 8 per group. Each dot represents an individual mouse; colors represent the experimental diet group. Mixed models with repeated measures showed a significant effect of group, with 15% HS consuming significantly more kilocalories than the 5% HS and control groups (*p* < 0.0001). Effect of week, *p* < 0.0001.

Body weight at baseline and endpoint and total body weight gain did not differ significantly among diet groups (Table 2). Total food intake was significantly higher in the 15% HS versus 5% HS and control groups (*p* = 0.0002). When analyzed using ANOVA with repeated measures, there was a significant increase in body weight across weeks (*p* < 0.0001), and this did not differ significantly among diet groups (Figure 3). Food and kilocalorie intake per g body weight gained was significantly higher in the 15% HS versus 5% and control groups (*p* < 0.0001; Figure 4 and Figure 5).

### 3.2. Body Composition

**Figure 6:**
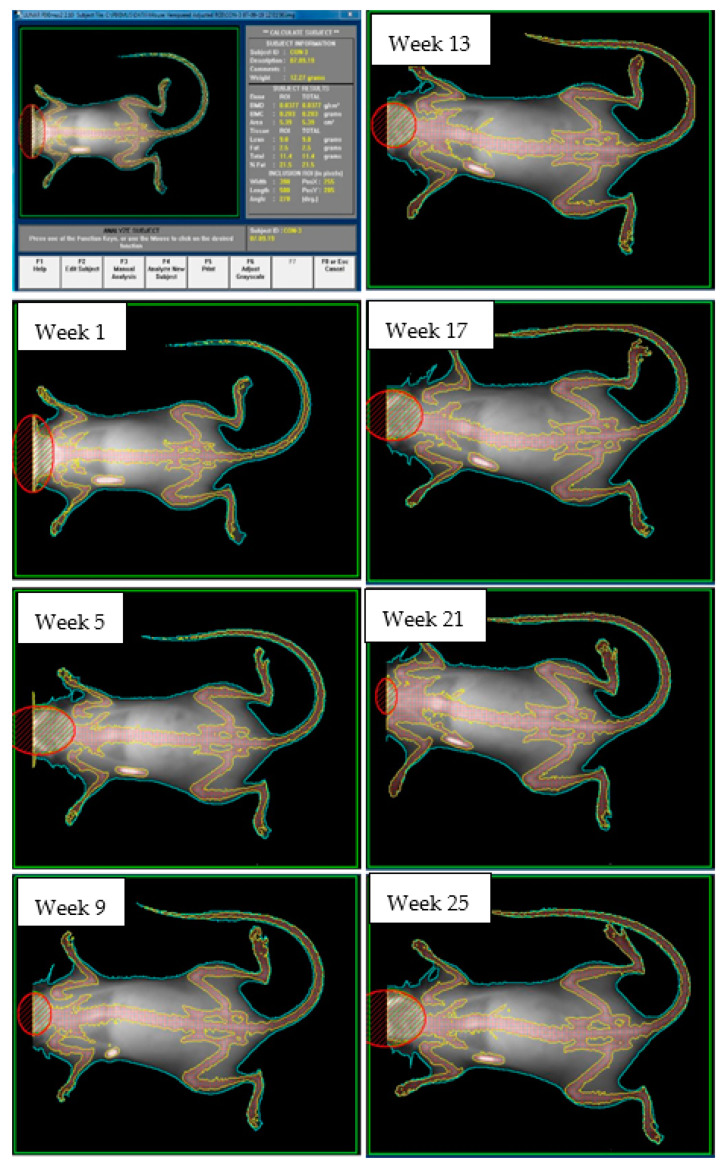
Representative time series of dual-energy X-ray absorptiometry images from one mouse (control group). Live mice were anesthetized and placed in the prone position for imaging. The skull was excluded from analysis. The white oval below the right forearm is an implanted identification microchip.

A representative time series of dual-energy X-ray absorptiometry images from one mouse (control group) is shown in Figure 6. The whole body with exclusion of the skull was selected for analysis and shows the progressive growth of mice observed from ages 5 to 30 weeks. The dense skeletal bones are marked by the perimeter yellow line for determination of bone area, bone mineral content (BMC) and bone mineral density (BMD). The less-dense body tissues (lean and adipose) include skin, muscles and visceral organs and these are located in the region bounded by the body perimeter line (blue) and skeletal bone line (yellow). In the prone position, the limbs could be extended perpendicular to the longitudinal axis. This ensured that area-dependent variables (BMD, g/cm^2^) were accurately measured. Density of the implanted microchip was not corrected for but remained constant throughout the experiment.

**Figure 7:**
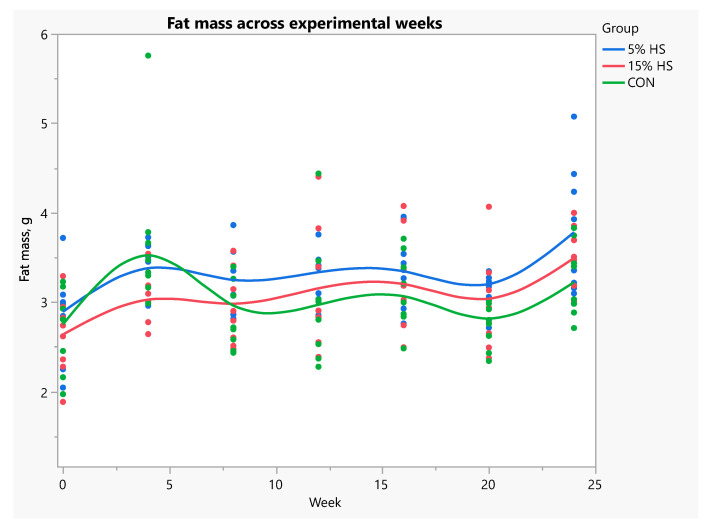
Fat mass (g) across experimental weeks. Measurements obtained by dual-energy X-ray absorptiometry in live mice. N = 8 per group. Each dot represents an individual mouse; colors represent the experimental diet group. Mixed models with repeated measures showed significantly higher fat mass in the 5% HS versus control diet group (*p* = 0.02). Effect of week, *p* = 0.002.

**Figure 8:**
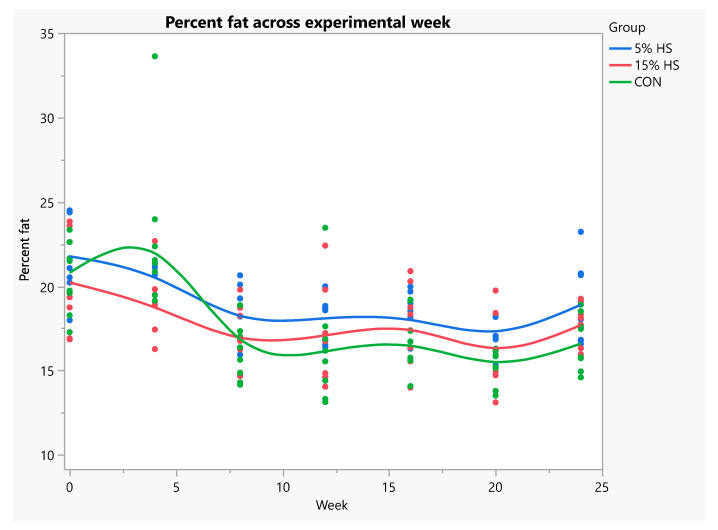
Percent body fat across experimental weeks. Measurements obtained by dual-energy X-ray absorptiometry in live mice. N = 8 per group. Each dot represents an individual mouse; colors represent the experimental diet group. Mixed models with repeated measures showed significantly higher percent fat in the 5% HS versus 15% HS and control diet groups (*p* = 0.03). Effect of week, *p* < 0.0001.

**Figure 9:**
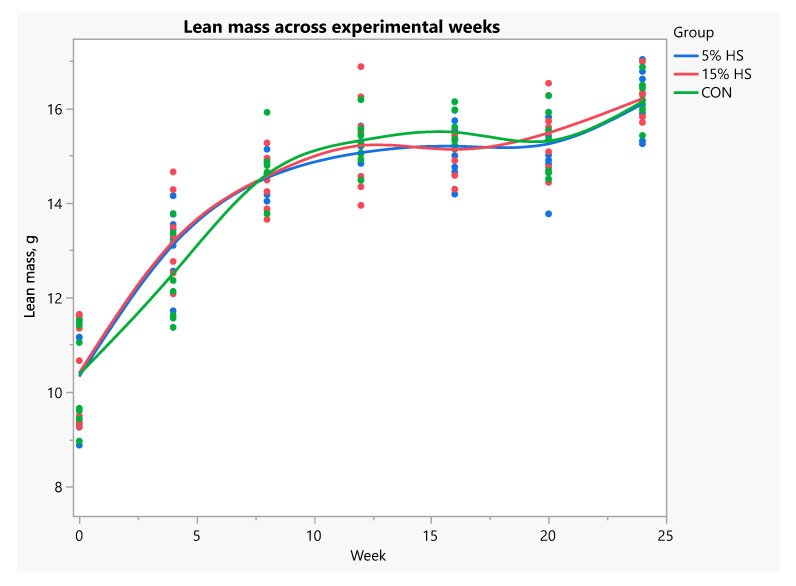
Lean mass (g) across experimental weeks. Measurements obtained by dual-energy X-ray absorptiometry in live mice. N = 8 per group. Each dot represents an individual mouse; colors represent the experimental diet group. Mixed models with repeated measures showed no significant differences among group (*p* = 0.92). Effect of week, *p* < 0.0001.

**Figure 10:**
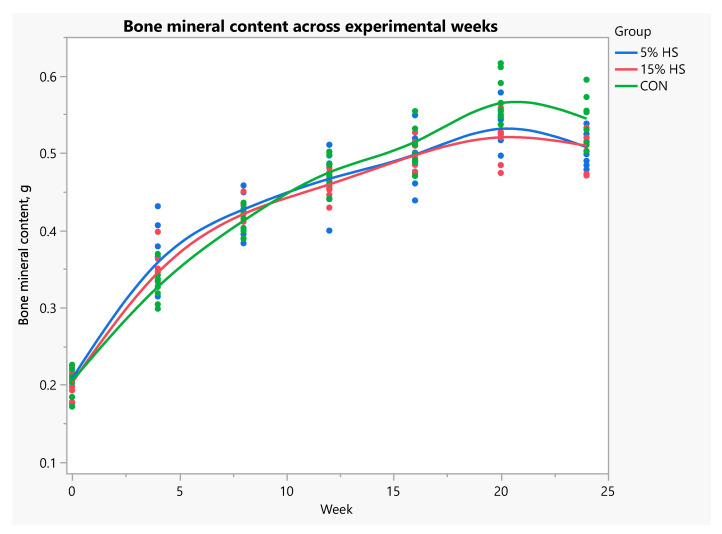
Bone mineral content (g) across experimental weeks. Measurements obtained by dual-energy X-ray absorptiometry in live mice. N = 8 per group. Each dot represents an individual mouse; colors represent the experimental diet group. Mixed models with repeated measures showed no significant differences among groups (*p* = 0.85). Effect of week, *p* < 0.0001.

When data were analyzed using repeated measures ANOVA, fat mass was significantly higher in the 5% HS versus control group across experimental weeks (*p* = 0.02; Figure 7). Percent fat across weeks was significantly higher in the 5% HS versus both the 15% HS and control groups (*p* = 0.03; Figure 8). Lean mass, bone mineral content and bone mineral density increased during the experiment (*p* < 0.0001), without a significant effect of group (Figure 9 and Figure 10).

**Figure 11:**
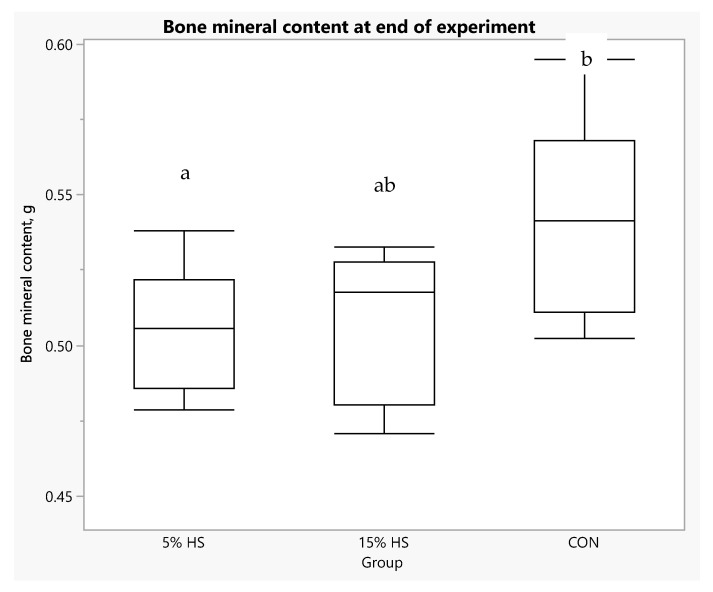
Box plot of bone mineral content (g) at the end of the experiment. Measurements obtained by dual-energy X-ray absorptiometry in live mice. N = 8 per group. Boxes depict: 75th percentile (upper edge), 50th percentile/median (middle line), 25th percentile (lower edge) and whiskers (maximum and minimum values). ANOVA main effect of group, *p* = 0.022. Boxes not sharing letter superscripts are significantly different.

When data were restricted to the final experimental week (week 25), one-way ANOVA showed higher values for fat mass and percent fat in the 5% HS compared to the other groups, but the differences did not reach statistical significance, (main effect of group, *p* = 0.12 and *p* = 0.059, respectively). Lean mass and BMD were very similar across groups (main effect of group, *p* = 0.94 and *p* = 0.72, respectively; Table 3). However, BMC at week 25 was significantly higher in the control compared to 5% HS group (*p* = 0.022; Figure 11).

### 3.3. Humerus Bone Osteoblast Cell Density, Mineralization and Growth Plate Thickness

**Figure 12:**
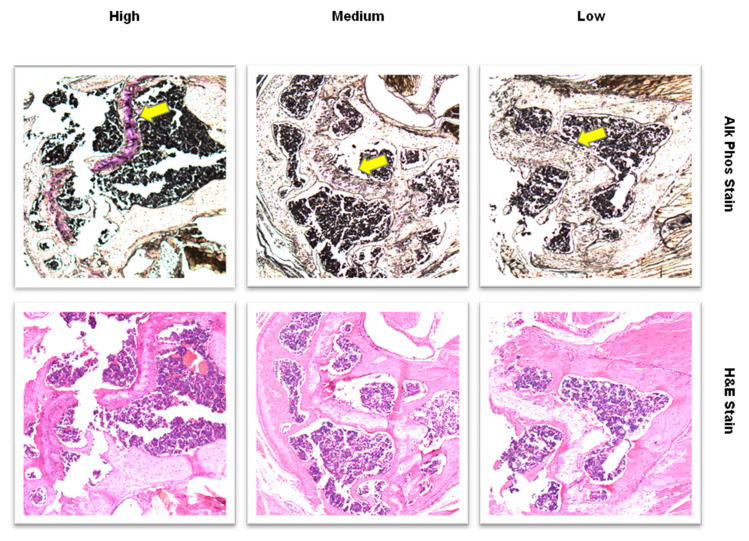
Histology images (10× magnification) of humerus sections at the distal epiphyseal plate from 3 mice. Purple areas identified with arrows show alkaline phosphatase staining of osteoblasts (top row). The images illustrate sections with high, medium and low staining. Hematoxylin and eosin-stained (H&E) images show cell structures within the sections (bottom row).

**Figure 13:**
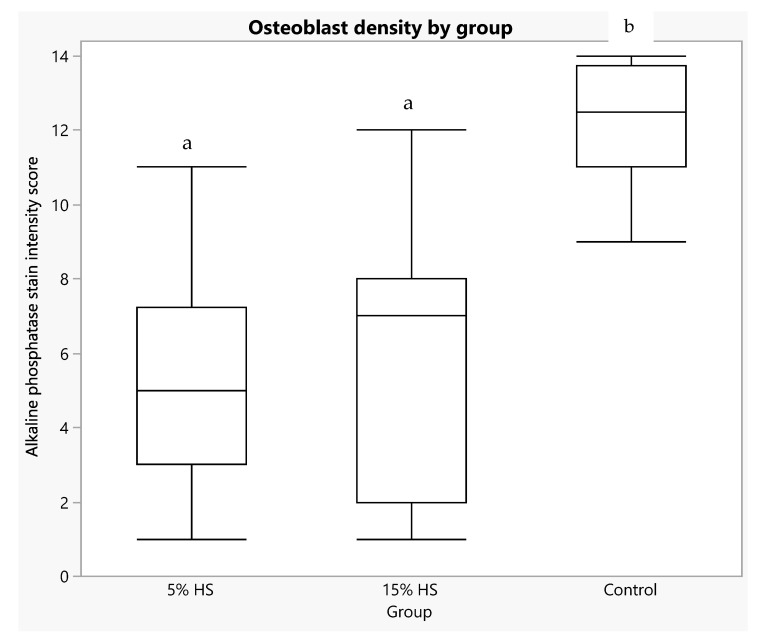
Box plot of alkaline phosphatase stain density score at the humerus epiphysis for each diet group. Higher scores equate to greater density of osteoblasts. Boxes depict: 75th percentile (upper edge), 50th percentile/median (middle line), 25th percentile (lower edge) and whiskers (maximum and minimum values). Main effect of group, *p* < 0.0001. Boxes not sharing letter superscripts indicate significantly different values.

All in vitro studies were performed on the left humerus bone at endpoint. Figure 12 shows three representative images of stained bone sections ranked as high, medium and low alkaline phosphatase density at the distal epiphyseal plate. Osteoblast density score at the humerus distal epiphyseal plate was significantly higher in the control (12.20 ± 1.58) compared to the 5% HS (5.40 ± 2.76) and 15% HS (5.95 ± 3.61) groups (*p* < 0.0001; Figure 13). A significant effect of group was also seen when comparing both HS groups with the control (*p* < 0.0001). No significant effect of scorer (among the five individuals who scored the alkaline phosphatase-stained images) was found (*p* = 1.00). No TRAP staining of osteoclasts was observed in the distal epiphyseal plate. Linear regression showed no significant relationship between osteoblast density score and site-specific, distal humerus bone values for BMC (R-squared = 0.02, *p* = 0.62) or BMD (R-squared = 0.16, *p* = 0.16) measured at week 25.

**Figure 14:**
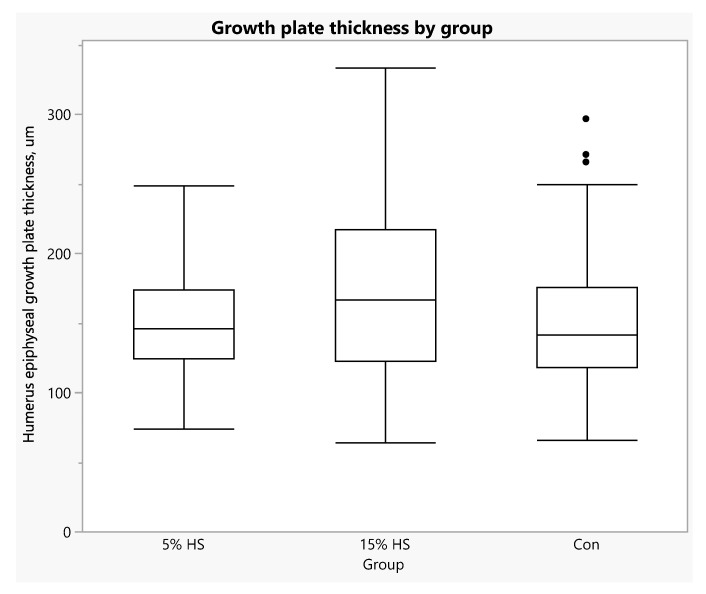
Box plot of thickness of the humerus epiphyseal growth plate for each diet group. Boxes depict: 75th percentile (upper edge), 50th percentile/median (middle line), 25th percentile (lower edge) and whiskers (maximum and minimum values). Main effect of group, *p* = 0.16. Removal of control group outliers (three dots above the 75th percentile line) resulted in a main effect of group of *p* = 0.06, with greater thickness in the 15% HS versus control group.

Even though there was higher alkaline phosphatase activity within the epiphyseal plate of the control versus the HS groups, there was no significant difference in the thickness of the growth plates across the three treatment groups (*p* = 0.16; Figure 14). Removal of the three outliers seen in Figure 13 from analysis changed the main effect of group to *p* = 0.06, with plate thickness being higher in the 15% HS compared to the control group. Growth plate thickness was not significantly related to osteoblast density score (R-squared = 0.05, *p* = 0.14).

### 3.4. PRDM16-Positive Cells in the Bone Marrow

**Figure 15:**
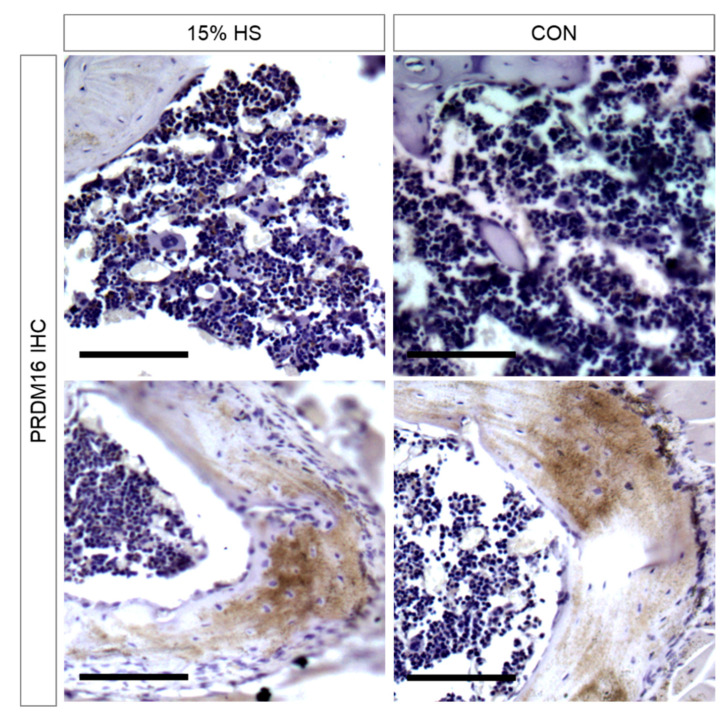
Humerus immunohistochemical staining of PRDM16-positive cells in representative mice from the 15% HS and control groups. Top row: Bone marrow sections showing the density of PRDM16-positive cells (brown color), an indication of differentiated adipocytes. Bottom row: Humerus metaphysis sections displaying comparable staining between the treatment groups. Scale bar indicates 100 μm.

As seen earlier in the fat mass analysis, the diet enriched with hempseed had the propensity to increase fat mass and percentage in the mice. Bone marrow was analyzed after immunohistochemical staining using an anti-PRDM16 antibody. PRDM16 is a zinc finger transcription factor and can interplay with the Smad3 and TGFb signaling pathways [48]. It has been shown to play a role in cell fate switching between skeletal myoblasts and fat cells [45,46,47]. Qualitatively, the mice that were treated with 15% HS had more PRDM16-positive cells in the bone marrow (Figure 15). Upon quantitative analysis of the percent PRDM16-positive cells in the bone marrow, the numbers confirmed that the HS-treated mice exhibited a higher percentage of PRDM16-positive cells. A total of 8015 cells were counted across control sections, 3727 cells were counted across 5% HS-treated sections, and 1924 cells were counted across 15% HS-treated sections. Respective percentages of PRDM16 positive cells were 10.0%, 14.5%, and 16.5%. Using a Chi-square test to compare the differences in observed versus expected/control, significant increases were determined at a *p* value < 0.0001. Further characterization of staining throughout the humeri demonstrated a concentration of staining in the metaphysis that was comparable between HS treated and control (Figure 15).

## 4. Discussion

This experiment examined the effects of hempseed-enriched diets (5% and 15% by weight) on body composition and bone parameters in growing female mice. Across the 25-week course of the study, body fat mass tracked significantly higher in the 5% HS group compared to the control and 15% HS groups and percent fat tracked higher in the 5% HS than the control group. However, at the end of the experiment, group differences in body fat measurements did not reach statistical significance. Lean mass was nearly identical among groups when examined across weeks and at endpoint.

Bone parameter measurements were affected by diet. Bone mineral content, considered to be a more appropriate index of bone mineralization than BMD (the ratio of BMC to bone size) in growing animals [49,50], was significantly lower in the 5% HS versus control group at the end of the experiment. Further, bone cell density measurements in one selected bone, the humerus, at the distal epiphyseal plate, revealed that osteoblast density was lower in both HS diet groups compared to the control. However, thickness of the epiphyseal growth plate and BMC measured by dual-energy X-ray absorptiometry were not significantly different across treatment groups.

The bone results were contrary to evidence supporting a beneficial effect of dietary hempseed on bone structure. Studies in hens and cockerels showed that hempseed-enriched diets increased tibia strength [35,36]. Hempseed feeding in rats significantly reduced liver oxidative stress as determined by thiobarbituric acid-reactive substances and increased antioxidant potential as measured by total glutathione levels [29]. Reactive oxygen species induce osteoclastogenesis and bone resorption [24] and diets high in omega-3 fatty acids, by means of enrichment with fish oil or flaxseed, result in reduced levels of pro-inflammatory cytokines [31,51]. Dietary supplementation with foods rich in α-linolenic acid (walnuts and flaxseed) versus linoleic-rich and “average American” diets for 6 weeks resulted in reduced levels of serum N-telopeptides of type I collagen, a marker of bone resorption, in association with reduced serum TNFα [8]. Further, peripheral blood mononuclear cells obtained from participants consuming the α-linolenic acid-rich diet compared to the other diets produced significantly lower concentrations of cytokines IL-6, IL-1β, and TNFα [52].

Differences in animal species and bone outcome measures may be factors contributing to our findings that differ from those of other investigators. We expected the antioxidant, anti-inflammatory properties of polyphenols and unsaturated fatty acids in the hempseeds to promote osteoblast activity, reduce osteoclast activity and increase bone mineralization. However, this was not the case, and the data instead suggest that a long-term hempseed diet may negatively impact new bone formation at the growth plate. Osteoblast density was reduced in mice fed a hempseed (versus control) diet. Tracking the time course of bone growth using in vivo labeling would provide important information on the effect of dietary hempseed on the rate of mineralization [53]. Notably, our measurement of humerus epiphyseal plate thickness at endpoint showed no effect of diet, suggesting that maturation of humerus longitudinal growth was not affected by dietary hempseed enrichment.

It is possible that phytochemical inhibitors in the hempseed diets interfered with mineral absorption [54], resulting in lower bone mineralization. Hempseed contains phytic acid, which forms insoluble complexes with minerals in the gastrointestinal tract [55,56,57,58]. Polyphenols, such as tannins, in hempseed may also inhibit the absorption of calcium and other minerals, although effects on bone structure are not consistent [59,60] and some studies show increases in calcium absorption with polyphenol supplementation [61]. Future studies employing fecal mineral analysis in conjunction with bone measurements could test this postulate. 

A general reduction in absorption and assimilation of the higher-concentration hempseed diet was demonstrated in this study by the significantly greater total food and kilocalorie intakes, yet similar measures of body weight and rate of weight gain, in the 15% HS versus 5% HS and control groups. Hempseeds may be similar to nuts in having a lower degree of digestibility than that calculated using Atwater general factors for macronutrient energy content [62,63]. Human studies demonstrate lower-than-predicted (by Atwater factors) metabolizable energy values from whole almonds, walnuts and pistachios [63,64,65]. Novotny et al. reported a 5% decrease in whole-diet energy digestibility when 84 g whole almonds were consumed daily [63]. The current study incorporated ground seeds into the hempseed diets, which may not have reduced diet digestibility as much as whole seeds. Metabolizable energy derived from nuts is affected by the form of the nut [66]. Mastication of intact nuts only partly disrupts cell wall structure to allow release of intracellular lipids for digestion [67,68]. Mechanical grinding likely ruptures seed/nut cell walls to a greater degree than chewing; therefore, the ground hempseed-enriched diets used in this study may have reduced diet digestibility by several percentage points. A feeding trial utilizing whole hempseeds might produce more pronounced effects on energy digestibility than observed in the current study. The higher fat mass and percent fat in the 5% HS versus control diet across experimental weeks may be due to an effect of hempseed components on lipid metabolism. Opyd et al. showed that rats fed a hempseed-enriched diet (12.5%) for 4 weeks displayed significant alterations in hepatic genes coding for transcription factors involved in lipid metabolism including peroxisome proliferator-activated receptor γ (PPARγ) [29]. PPARγ binds multiple polyphenols and fatty acids found in hempseed [69] and the 5% HS diet in the current study may have provided ligands that modulated PPARγ activity. The reason that the 15% HS diet did not result in as large of a difference (compared to controls) in fat mass and body fat percentage as the 5% HS group might reflect lower bioavailability of polyphenols and fatty acids in the 15% HS diet.

The finding of greater PRDM16 staining in epiphyseal marrow of the 5% and the 15% HS compared to control group is suggestive of a relative increase in stem cell differentiation into adipogenic over osteogenic lineages in the HS mice. However, staining did not appear to differ across groups when examining the metaphyseal humerus. High adipocyte density in bone marrow is associated with poorer bone integrity [23,33,34]. Bone marrow adipocytes can comprise between 50 and 70% of all bone marrow cells. In the present study, we evaluated only the presence of brown adipocytes because of their role in enhancing energy expenditure [70]. Prior studies report a beneficial effect of dietary omega-3 fatty acids and polyphenols on bone marrow cell differentiation to osteoblasts and on bone density [16,17,20,23,27]. The significance of our findings is uncertain and additional data that address impacts on bone strength of long bones and vertebrae, especially at older ages, are needed before reaching conclusions.

Strengths of this study include the long-duration feeding of hempseed diets during the growth period of mice, which provided presumably adequate exposure to the treatment for effects to be observed. The monthly dual-energy X-ray absorptiometry measurements allowed for the monitoring of changes in body composition across the 6-month experiment. Additionally, the use of whole hempseeds for diet enrichment replicates the consumption of hempseed within a whole-foods diet in humans. This is important for describing the health effects of whole hempseed consumption, which captures outcomes of the interactions of dietary nutrients within a food within a diet. A limitation is the inclusion of only female mice. Future investigations should include male animals along with the extension of the feeding intervention through older age in order to determine the effects of habitual hempseed consumption on age-related bone loss.

## 5. Conclusions

Our findings demonstrate that long-term dietary enrichment with hempseed impacts the trajectory of body fat content across time and endpoint measures of bone mineral content and osteoblast density. The results do not support a beneficial effect of hempseed consumption on bone mineralization or bone marrow cell populations.

## Figures and Tables

**Table 1:** Composition of experimental diets.

Ingredient, g per kg Diet	Control Diet ^a^	5% Hempseed	15% Hempseed
Casein, High Nitrogen	200	185	155
L-Cystine	3	3	3
Sucrose ^b^	100	100	100
Cornstarch	397.486	395.99	392.997
Dyetrose	132	132	132
Soybean Oil	70	52	16
t-Butylhydroquinone	0.014	0.01	0.003
Cellulose	50	34.5	3.5
Mineral Mix #210025 ^c^	35	35	35
Vitamin Mix #310025 ^d^	10	10	10
Choline Bitartrate	2.5	2.5	2.5
Hempseed ^e^	0	50	150
Total	1000	1000	1000
Kilocalories per kg	3760	3814	3922

^a^ AIN-93G. ^b^ Ninety percent tetrasaccharides and higher; ^c^ Composition (g/kg mineral mix): CaCO_3_, 357.0; KH2PO_4_, 196.0; K Citrate•H_2_O, 70.78; NaCl, 74.0; K_2_SO_4_, 46.6; MgO, 24.3; Fe citrate, 6.06; ZnCO_3_, 1.65; MnCO_3_, 0.63; CuCO_3_, 0.31; KIO_3_, 0.01; Na_2_SeO_4_, 0.01025; (NH_4_)_6_ Mo_7_O_24_•4H_2_O, 0.00795; Na_2_SiO_3_•9H_2_O, 1.45; CrK(SO_4_)_2_•12H_2_O, 0.275; LiCl, 0.0174; H_3_BO_3_, 0.0815; NaF, 0.0635; 2NiCO_3_•3Ni(OH)_2_•4H_2_O, 0.0318; NH_4_VO_3_, 0.0066. ^d^ Composition (g/kg vitamin mix): thiamin HCl, 0.6; riboflavin, 0.6; pyridoxine HCl, 0.7; nicotinic acid, 3.0; Ca pantothenate, 1.6; folic acid, 0.2; D-biotin, 0.02; vitamin B^12^ (0.1% in mannitol), 2.5; vitamin A palmitate (500,000 IU/g), 0.8; DL-α-tocopheryl acetate (500 IU/g), 15; vitamin D3 (400,000 IU/g), 0.25; vitamin K/dextrose 10 mg/g (phylloquinone), 7.5. ^e^ Whole ground hempseed (nutrition information supplied in Supplementary Table S1).

**Table 2:** Body weight at baseline and endpoint and total weight gain and food intake.

Group	Baseline Body Weight (g)	Endpoint Body Weight (g)	Total Body Weight Gain (g)	Total Food Intake (g)
Control	14.60 (0.79)	21.89 (0.58)	7.29 (0.60)	454.37 (14.38) *^a^
5% HS	14.64 (1.09)	21.66 (1.09)	7.02 (1.09)	448.17 (6.68) ^a^
15% HS	14.72 (0.61)	21.37 (0.80)	6.65 (0.85)	475.30 (12.17) ^b^

Values are means with standard deviation in parentheses. N = 8 per group. * *p* = 0.0002 main effect of group on food intake. Values within columns that share superscript letters are not significantly different.

**Table 3:** Body composition at end of experiment.

Group	Fat Mass (g)	Percent Fat (%)	Lean Mass (g)	BMD (g/cm^2^)	BMC (g)
Control	3.25 (0.41)	16.69 (1.64)	16.18 (0.43)	0.056 (0.002)	0.541 (0.033) ^a^
5% HS	3.81 (0.72)	18.99 (2.40)	16.12 (0.66)	0.055 (0.002)	0.505 (0.021) ^b^
15% HS	3.52 (0.33)	17.81 (1.17)	16.21 (0.41)	0.055 (0.003)	0.508 (0.024) ^ab^

Values are means with standard deviation in parentheses. N = 8 per group. One-way ANOVA within columns showed a significant effect of group only for BMC, *p* = 0.022. Values with the column not sharing letter superscripts are significantly different. BMD = Bone Mineral Density; BMC = Bone Mineral Content.

## Data Availability

The data presented in this study are available on request from the corresponding author.

## Supplementary Materials

The following supporting information can be downloaded at: https://www.mdpi.com/article/10.3390/ijerph19105839/s1, Table S1: Nutrition information for hempseed.
