# Supplementary material for: The Impact of Hempseed Consumption on Bone Parameters and Body Composition in Growing Female C57BL/6 Mice
Source: Int J Environ Res Public Health. 2022 May 11;19(10):5839. doi: 10.3390/ijerph19105839 (PMC9140819; doi:10.3390/ijerph19105839)
Supplement: Supplementary file 1 [file ijerph-19-05839-s001.zip › ijerph-1692029-supplementary.pdf]

Supplementary Table S1. Nutrition information for hempseed

Amount per 100 g	
Kilocalories	550
Total fat, g	36
Polyunsaturated fat, g	27
Omega-6 fat, g	20
Omega-3 fat, g	7
Monounsaturated fat, g	4
Saturated fat, g	3
Carbohydrate, g	31
Dietary fiber, g	29
Soluble fiber, g	2
Insoluble fiber, g	27
Protein, g	26
Sodium, mg	0
Potassium, mg	760
Calcium, mg	110
Iron, mg	13
Vitamin E, mg	2.2
Thiamin, mg	0.6
Riboflavin, mg	0.3
Niacin, mg	4
Vitamin B6, mg	0.7
Folate, mg	0.16
Phosphorus, mg	960
Magnesium, mg	430
Zinc, mg	6.7
Copper, mg	1.7
Manganese, mg	12

Information provided by Natura™ for CHII whole toasted hempseed; https://natera.ca/product-category/chii-hemp/ (accessed on 7 May 2022)
